# Flow Characteristics and Enhanced Oil Recovery Performance of Anionic and Zwitterionic Viscoelastic Surfactant System

**DOI:** 10.3390/gels11080627

**Published:** 2025-08-10

**Authors:** Chenyue Ling, Yafei Liu, Xuchun Yang, Qi Ye, Desheng Zhou

**Affiliations:** 1 College of Petroleum Engineering, Xi’an Shiyou University, Xi’an 710065, China; 23212010108@stumail.xsyu.edu.cn (C.L.); 24212010117@stumail.xsyu.edu.cn (X.Y.); dshzhou@xsyu.edu (D.Z.); 2 Oil Production Plant No.5 of Changqing Oilfield, China National Petroleum Corporation, Xi’an 710201, China; yq3_cq@petrochina.com.cn

**Keywords:** viscoelastic surfactant mixture, oil–water two-phase flow, microfluidics, enhanced oil recovery

## Abstract

Surfactant flooding has shown potential in enhanced oil recovery (EOR), but conventional surfactants often underperform in heterogeneous reservoirs. This study investigates the impact of a surfactant mixture, combining anionic sodium dodecyl sulfate (SDS) and zwitterionic oleylamidopropyl betaine (OAB-30), on two-phase flow behavior and its EOR potential. Six surfactant solutions with varying concentrations were first screened using an idealized dead-end shaped microchannel in combination with interfacial properties and rheological tests. The results showed that 0.2% SDS and 0.6% OAB-30 produced the highest oil recovery in the dead-end structure. Interfacial tension was reduced to 0.374 mN/m and strong viscoelastic behavior was observed using the optimized surfactant mixture. Wettability of the surface tended to be more hydrophilic after the application of the surfactant mixture as well. Subsequently, the microscale oil displacement process was examined using the optimized surfactant mixture via microfluidic devices with an idealized pore–throat network with permeability contrast and realistic pore–throat structure. The application of the optimal surfactant formula resulted in 28.46% and 49.96% improvement over conventional water flooding in a realistic pore–throat structure and idealized pore–throat network. The critical micelle concentration measurements of the mixture suggested favorable micelle formation, contributing to gel-like properties that improved sweep efficiency by lowering the mobility ratio. In heterogenous pore–throat networks, the emulsification, micellar solubilization, wettability alteration, and viscoelastic properties of the surfactant mixture favored the oil recovery process. This work provides experimental evidence and mechanistic insights for the application of viscoelastic surfactants in EOR in heterogeneous reservoirs.

## 1. Introduction

Waterflooding, attributed to its accessibility and cost-effectiveness, has become the most extensively utilized oil recovery method [1]. However, the difference in viscosity between water and crude oil has resulted in an irregular oil front diminishing sweep efficiency [2]. Moreover, relatively higher interfacial tension between oil and water leads to higher capillary resistance, which in turn results in a substantial amount of residual oil within the reservoir [3]. Given the limitations of waterflooding, chemical enhanced oil recovery [4,5,6,7,8], particularly surfactant flooding, is increasingly being employed to improve the oil recovery [9]. Surfactant flooding is capable of reducing the flowing resistance of crude oil and enhancing sweep efficiency by lowering the interfacial tension between oil and water and altering the wettability [10,11].

Conventional single-surfactant systems have encountered limitations in practical applications. For example, anionic surfactants such as sulfonates [12] and sulfates [13], as well as nonionic surfactants [14], exhibit inadequate salt and temperature tolerance. In high-salinity and high-temperature reservoir environments, these surfactants experience a significant decline in chemical stability. In contrast, cationic surfactants are prone to substantial adsorption losses on sandstone reservoir rock surfaces [15]. This results in the consumption of a considerable amount of active surfactant components during the displacement process. Furthermore, the dynamic interfacial behavior of single-surfactant systems is challenging to adapt to the displacement process under complex reservoir conditions [16,17]. To surmount the limitations of single-surfactant systems, researchers have proposed the application of surfactant mixtures. The primary types encompass anionic–anionic [18], anionic–cationic [19], anionic–zwitterionic [20], and anionic–nonionic [21] surfactant mixtures. Among these combinations, viscoelastic surfactant (VES) formed by combining anionic and zwitterionic surfactants exhibits strong synergistic effects, significantly enhancing solution viscosity, surface activity, and thermal/salinity tolerance, thus making it ideal for complex applications [22,23]. Some of these mixtures can form wormlike micelles and entangled structures in aqueous solutions through hydrophobic interactions, electrostatic interactions, and hydrogen bonding, resulting in viscoelastic solutions [24]. Viscoelastic surfactants possess unique rheological properties that enable the regulation of the oil displacement process at the microscopic scale. Their viscoelasticity can effectively suppress fingering during the displacement process and improve the uniformity of fluid flow in the reservoir [25]. Solid understanding of the EOR potential and oil displacement mechanism of these surfactant mixtures is necessary for a broader application of surfactants.

In recent years, numerous studies have utilized macro-scale displacement techniques and numerical simulations to assess the effectiveness of various displacement fluids [26,27,28]. However, research concerning the interfacial phenomena at the micro-scale is still insufficient. To gain a more comprehensive understanding of the underlying mechanisms governing the behavior of displacement fluids, investigations into the micro-scale interfacial dynamics are critical. Therefore, microfluidic approaches have been employed for advancing the understanding of these mechanisms at the microscale. It is distinguished by its ability to visualize fluid flow dynamics and replicate the pore–throat architecture of reservoir rock. These attributes render it an invaluable tool for unraveling the intricacies of how surfactant mixtures improve oil recovery efficiency [29]. Microfluidic devices are usually fabricated with a network of grooves or microchannels etched into diverse substrates, including glass [30], silicon, and polymers such as polydimethylsiloxane (PDMS) and polymethylmethacrylate (PMMA) [31,32]. Microfluidic technology has been widely applied in the field of EOR. For instance, realistic sandstone micromodels were fabricated to scrutinize the oil displacement efficacy of a novel zwitterionic Gemini surfactant [33]. A microfluidic device with an idealized pore–throat structure was applied to delineate the spontaneous imbibition phenomena occurring within distinct pore–throat configurations [34]. While previous research has predominantly focused upon the assessments of fluid properties and theoretical computations [35,36,37], it is still necessary to incorporate microscopic experimental investigations for a comprehensive understanding on the performance of varying surfactant mixtures. Extensive research has been conducted on surfactant mixtures including viscoelastic surfactants. Existing literature mostly demonstrated their positive effects at the macro-scale, particularly in enhancing recovery rates. However, limited attention has been paid to the micro-scale interfacial phenomena and the underlying oil displacement mechanisms. Therefore, microfluidic technology was employed in this study to investigate the micro-scale behavior of viscoelastic surfactants. Specifically, the study examined the interactions of sodium dodecyl sulfate (SDS) and oleylamidopropyl betaine (OAB-30) with various concentration ratios. Interfacial tension, viscoelasticity, viscosity, and contact angle were measured to conduct a comprehensive evaluation of the surfactant mixture’s characteristics and its performance on the oil recovery. Subsequently, the oil recovery mechanism of the optimal surfactant formulation was investigated using micromodels with varying pore–throat structures.

## 2. Results and Discussion

### 2.1. Fluid Properties of Surfactant Mixtures

#### 2.1.1. Interfacial Tension

**Figure 1:**
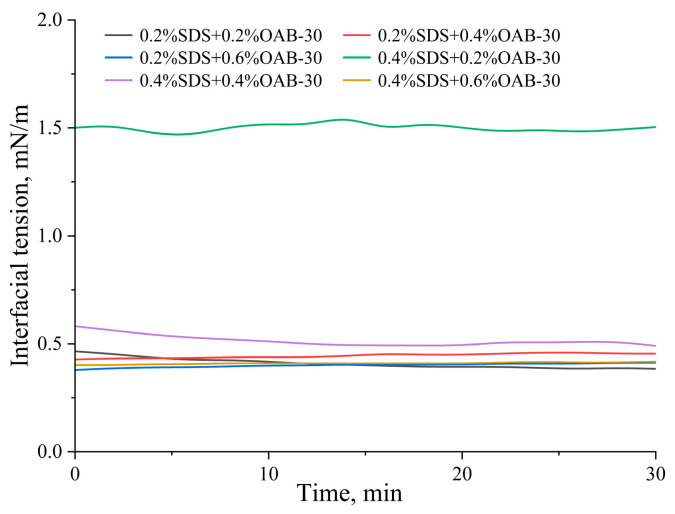
Dynamic interfacial tension of varying surfactant mixtures versus time.

Interfacial tension measurements of the six surfactant formulations revealed that the 0.2% SDS+0.2% OAB-30 solution achieved the lowest interfacial tension, closely followed by the 0.2% SDS+0.6% OAB-30 formulation. In contrast, the 0.4% SDS+0.2% OAB-30 solution exhibited the highest interfacial tension among all tested combinations. The complete interfacial tension profiles are presented in Figure 1, showing the dynamic changes during the measurement period.

Table 1 shows the interfacial tension data between crude oil and varying surfactant mixtures measured in this study.

#### 2.1.2. Viscosity

**Figure 2:**
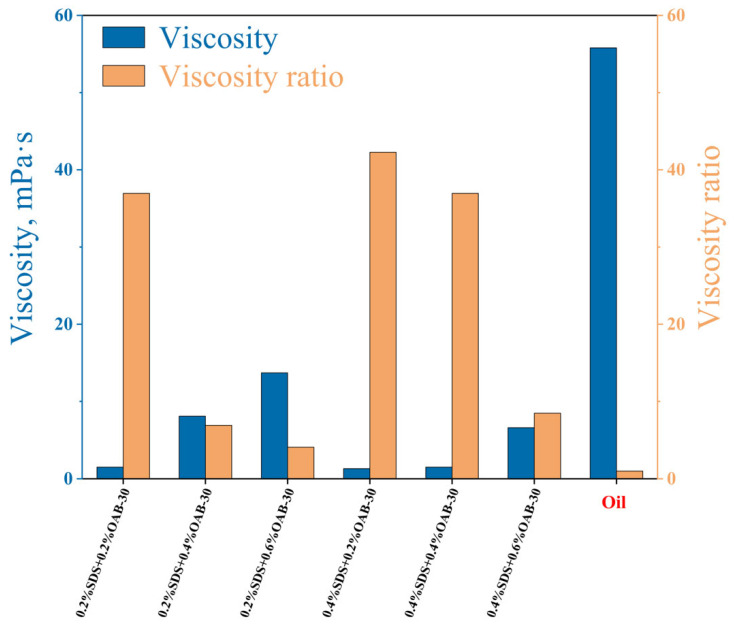
Viscosity of crude oil and surfactant mixtures with different concentrations and viscosity ratio between oil and the surfactant solutions.

Viscosity represents a fluid’s internal frictional resistance to flow deformation that reflects the hindrance of molecular interactions to fluid motion. As shown in Figure 2, at a temperature of 30 °C and a shear rate of 170 rpm, the blended oil (crude oil + liquid paraffin) exhibited a viscosity of 55.8 mPa·s. The results showed that surfactant solution with 0.2% SDS+0.6% OAB-30 displayed the highest viscosity with a 1:4 ratio to the crude oil viscosity.

#### 2.1.3. Contact Angle

**Figure 3:**
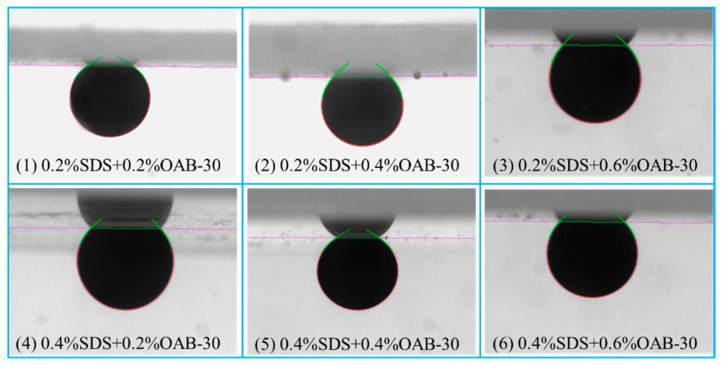
Images of contact angle measurement using different surfactant mixtures.

**Figure 4:**
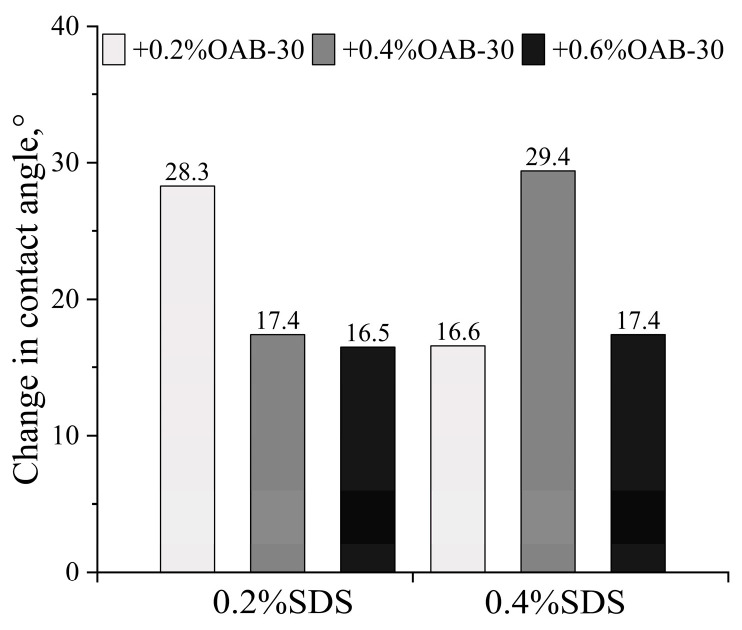
The change in the contact angle for different surfactant mixtures.

The initial contact angle measured after crude oil aging was 64.6 ± 2.38°. As shown in Figure 3 and Figure 4, all surfactant mixtures resulted in wettability alteration towards a more hydrophilic state. The maximum wettability alteration occurred when SDS and OAB-30 were mixed at a 1:1 concentration ratio. The 0.2% SDS+0.6% OAB-30 formulation (1:3 ratio) showed the least effective wettability alteration among all tested solutions.

#### 2.1.4. Viscoelasticity

**Figure 5:**
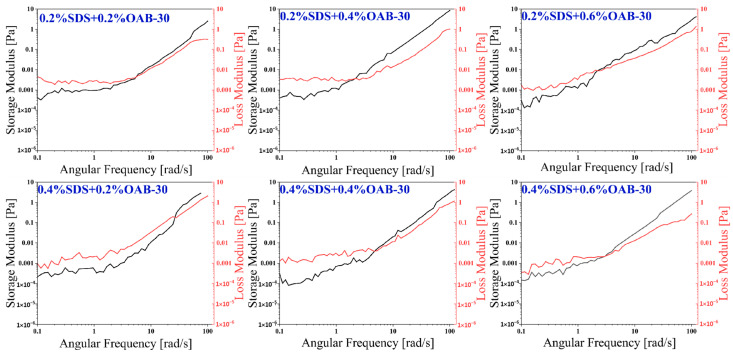
Storage modulus (G’) and loss modulus (G’’) versus angular frequency for different surfactant mixtures.

Rheological measurements were conducted to characterize the viscoelastic properties of each formulation. Analyses of the six sets of angular frequency versus storage modulus (G’) and loss modulus (G’’) curves are presented in Figure 5. At a fixed concentration of SDS, an increase in the concentration of OAB-30 results in a decrease in the cross-over angular frequency and an increase in the relaxation time and an enhancement in elasticity. Conversely, at a fixed concentration of OAB-30, an increase in the concentration of SDS results in an increase in the cross-over angular frequency, a decrease in the relaxation time and a relatively greater enhancement in viscosity. The 0.2% SDS+0.6% OAB-30 and 0.4% SDS+0.6% OAB-30 solutions exhibit a plateau region with a relatively high plateau modulus, which indicates a stronger elastic network. The viscoelastic properties of both groups are relatively good overall, with the 0.2% SDS+0.6% OAB-30 group exhibiting better elasticity at lower SDS concentrations. The enhanced viscoelasticity of the formulated solution indicates improved structural integrity and increased flow resistance. These characteristics are essential for maintaining effective mobility control during oil displacement processes.

### 2.2. Screening of the Optimal Surfactant Formula

**Figure 6:**
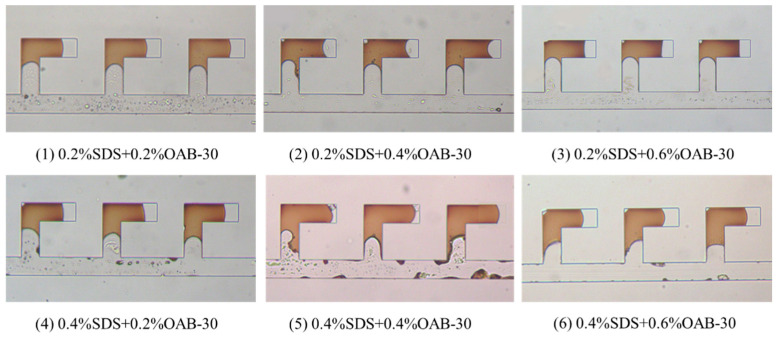
Final image after displacement process in the L-shaped dead-end pore structure using different surfactant mixtures.

Figure 6 presents the results of displacement experiments in dead-end pores utilizing six distinct surfactant formulations. Quantitative analysis was performed using Image J software (NIH, Win64) to determine the respective oil recovery efficiencies. The final recovery rates were calculated by averaging results from three central L-shaped structures for each formulation.

**Figure 7:**
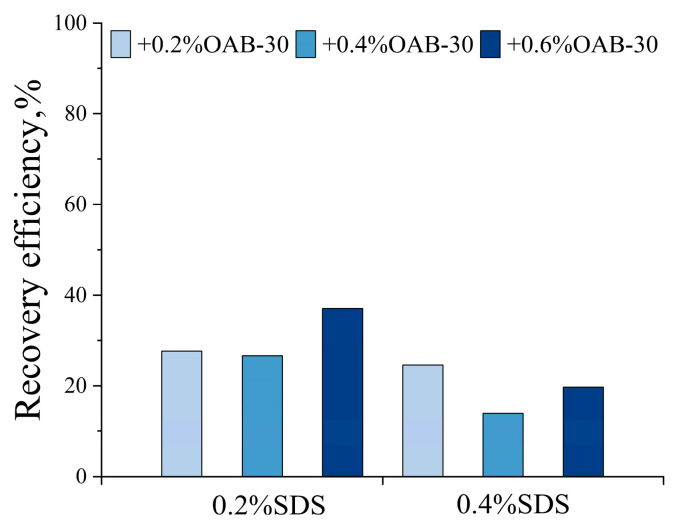
Oil recovery efficiency in L-shaped dead-end pore structure for different surfactant solutions.

As illustrated in Figure 7, displacement tests in the dead-end pores revealed that the optimal performance was achieved by the 0.2% SDS+0.6% OAB-30 solution, which demonstrated a superior recovery rate of 37.10%.

Fluid property measurements revealed that the 0.2% SDS+0.6% OAB-30 solution exhibited the highest viscosity with a favorable 1:4 viscosity ratio to crude oil. This elevated viscosity ratio could potentially suppress viscous fingering and promote stable displacement fronts with uniform advancement. In addition, this system achieved relatively lower interfacial tension, favorable viscoelastic properties, and wettability alteration. The relatively high viscoelasticity improves the rheological properties of the displacement fluid, enabling a more uniform flow field within the pores. This could potentially reduce fluid fingering and enhance sweep efficiency. Additionally, even with limited changes in wettability, the adhesive force between oil droplets and pore walls can be reduced, thereby improving oil mobility. The 0.2% SDS+0.6% OAB-30 surfactant mixture resulted in efficient oil displacement through synergistic effects: IFT reduction, wettability modification and viscoelastic flow control. These synergistic effects collectively contributed to its optimal oil recovery performance. Consequently, this formulation was selected as the optimized surfactant formula for subsequent displacement experiments.

### 2.3. Microscale Displacement Experiments Using Two Different Pore–Throat Structures

**Figure 8:**
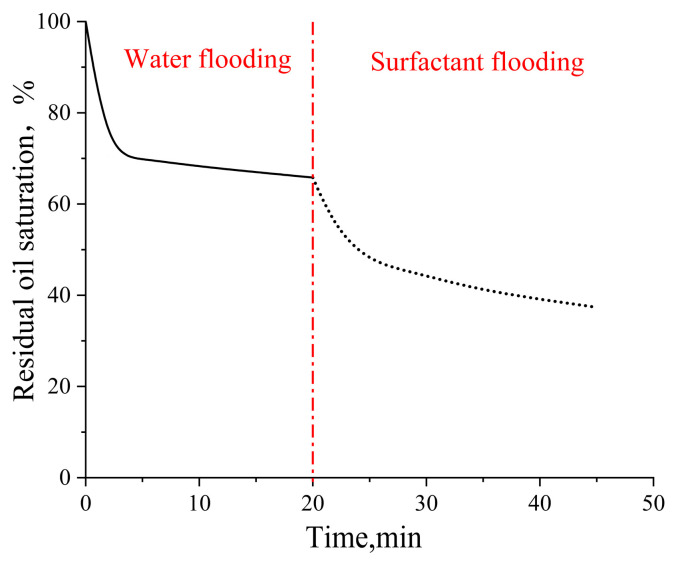
Residual oil saturation change in the pore–throat structure emulating realistic reservoir rock. Solid line represents the waterflooding stage and dashed line represents the surfactant flooding stage.

In the pore–throat structure emulating realistic reservoir rock, water flooding achieved a recovery factor of 34.18% as shown in Figure 8. Subsequent surfactant flooding significantly enhanced the oil recovery up to 62.64%, demonstrating a remarkable 28.46% improvement over conventional water flooding.

**Figure 9:**
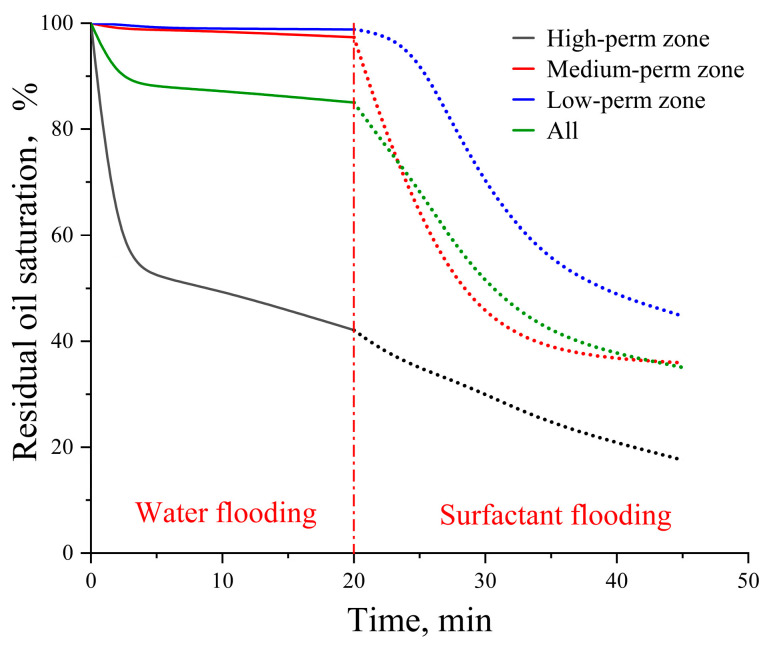
Residual oil saturation change in the idealized pore–throat network with permeability contrast. Solid lines represent the waterflooding stage and dashed lines represent the surfactant flooding stage.

For the idealized pore–throat network with permeability contrast, conventional formation water flooding resulted in residual oil saturations of 42.11% in the high-permeability (High-perm) zone, 97.36% in the medium-permeability (Medium-perm) zone, and 98.82% in the low-permeability (Low-perm) zone as shown in Figure 9. Subsequent surfactant flooding using the optimized surfactant solution significantly reduced these values to 17.57%, 35.93%, and 44.76%, respectively, indicating effective oil mobilization from both medium and low permeability zones. The overall final oil recovery rate was enhanced by 49.96% compared to that achieved by formation water flooding.

When comparing the recovery rates in the two structures, structural variability leads to different degrees of recovery enhancement. In the pore–throat structure emulating realistic reservoir rock, the pore–throat topology is complicated, possessing tortuous channels and differently shaped dead-end pores. On the other hand, for the idealized pore–throat network with permeability contrast, the pore–throat space is regular and there is no dead-end space. Therefore, the enhancement over conventional waterflooding by the surfactant mixture is lower in the pore–throat structure emulating realistic reservoir rock.

The experimental results have demonstrated the superior performance of anionic and zwitterionic surfactant mixtures in enhanced oil recovery. Table 2 shows various anionic–zwitterionic surfactant mixtures and their respective increases in oil recovery efficiency in the published work. Overall, the combination of anionic and zwitterionic surfactants demonstrates excellent effectiveness in enhancing oil recovery for various systems.

### 2.4. Oil Displacement Mechanism of the Optimized Surfactant Mixture

In an oil–water two-phase system, the addition of surfactants leads to the formation of mixed adsorption layers at the interface with the lipophilic groups penetrating into the oil phase and the hydrophilic groups in the aqueous phase. This configuration transforms the originally high-energy oil–water interface into a low-energy surfactant adsorption layer, thus effectively mitigating interphase repulsion forces and achieving significant reduction in interfacial tension.

**Figure 10:**
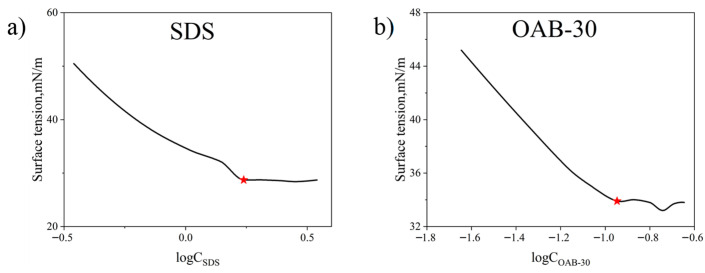
Measured surface tension versus varying surfactant concentrations for (**a**) SDS and (**b**) OAB-30.

The combination of SDS and OAB-30 facilitates the formation of micellar networks, leading to the development of gel-like substances. This endows the system with superior viscoelastic properties, thereby enhancing the ability to mobilize and transport crude oil. The critical micelle concentration (CMC) values for individual surfactants were determined through the surface tension measurements, and the results are presented in Figure 10. The CMCs for SDS and OAB-30 are 1.73 and 0.11 mmol/L respectively which are marked with red stars in Figure 10.

**Figure 11:**
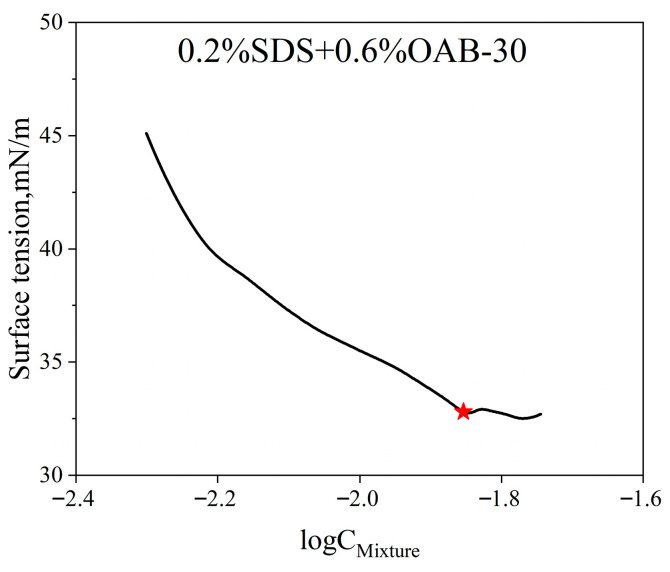
Measured surface tension versus log surfactant mixture concentrations of the surfactant mixture.

The CMC of the surfactant mixture was determined using the surface tension method as well. As shown in Figure 11, the measured CMC for the surfactant mixture is 0.014 mmol/L which is marked with the red star.

According to the Rubingh theory, for two different surfactants forming mixed micelles in an aqueous phase, the interaction parameter β^M^ in the mixed micelles can be calculated using the following equations:

(1) $$\frac{{\left(X_{1}^{M}\right)}^{2}ln\left(\alpha_{1}C_{12}^{M}/X_{1}^{M}C_{1}^{M}\right)}{{\left(1-X_{1}^{M}\right)}^{2}ln\left[\left(1-\alpha_{1}\right)C_{12}^{M}/\left(1-X_{1}^{M}\right)C_{2}^{M}\right]}=1$$

(2) $$\beta^{M}=\frac{ln\left(\alpha_{1}C_{12}^{M}/X_{1}^{M}C_{1}^{M}\right)}{{\left(1-X_{1}^{M}\right)}^{2}}$$

where X_1_^M^ represents the molar fraction of surfactant 1 in the mixed micelles, and β^M^ is a parameter that characterizes the nature and extent of the interaction between the two different surfactants in the mixed micelles; α_1_ is the molar fraction of SDS in the solution with a value of 0.34. C_12_^M^ is the CMC of the 0.2% SDS+0.6% OAB-30 surfactant mixture. C_1_^M^ and C_2_^M^ are the CMCs of SDS and OAB-30, respectively. By iteratively solving Equation (1) to obtain the value of X_1_^M^, the interaction parameter β^M^ can be evaluated using Equation (2). A positive β^M^ value indicates antagonistic interactions in the mixed system, while a negative β^M^ value suggests synergistic interactions [42]. The calculated value of β^M^ is −13.2, indicating that the combination of the two surfactants exhibits a synergistic effect.

**Figure 12:**
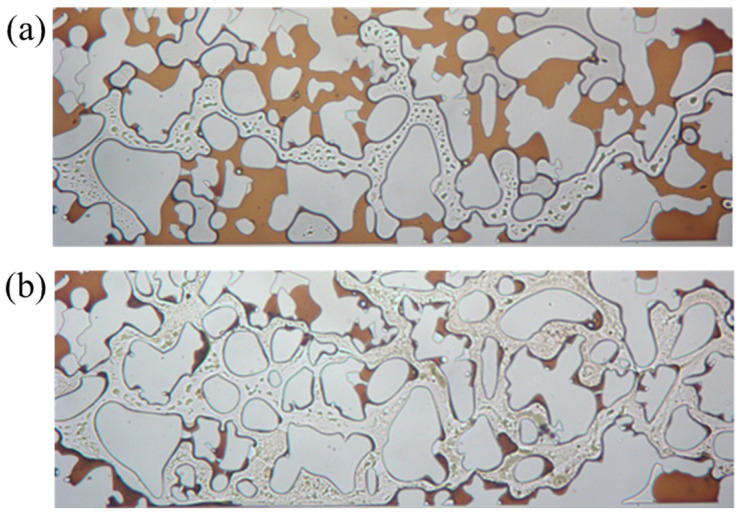
Sweep comparison (**a**) before and (**b**) after surfactant flooding.

Figure 12 reveals that in the pore–throat structure emulating realistic reservoir rock, the substantial heterogeneity in flow resistance across different pore networks leads to the development of preferential flow paths through well-connected macropores during conventional waterflooding. This results in significant bypassing of oil trapped in poorly connected dead-end pores and narrow throat channels. Substantial residual oil saturation remained unrecoverable through standard water injection methods. Upon switching to surfactant solution flooding, a remarkable improvement in displacement efficiency was observed. The swept area expands substantially, and previously unrecoverable oil by waterflooding was rapidly mobilized and displaced. This pronounced enhancement in recovery performance directly results from the surfactant mixture’s ability to reduce interfacial tension, alter wettability, and improve mobility control. Therefore, the capillary trapping that constrains waterflood effectiveness in heterogeneous pore structures was overcome. The visual evidence confirms the superior oil mobilization capability of the viscoelastic surfactant solutions in complex porous media.

**Figure 13:**
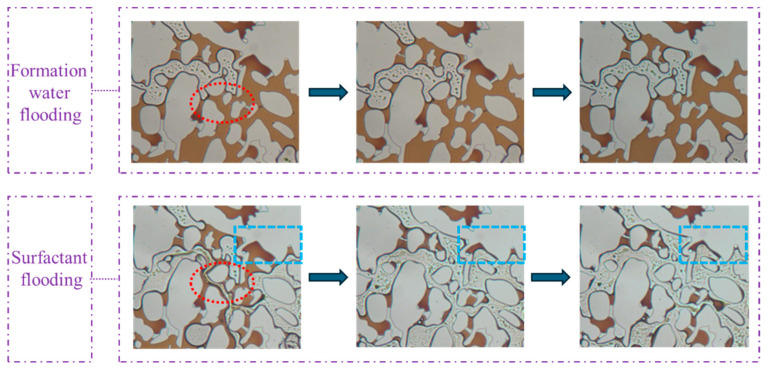
Images of the flooding experiment using formation water and surfactant in the pore–throat structure emulating realistic reservoir rock.

The observations from Figure 13 indicate that the regions circled by blue dotted lines demonstrate the surfactant system’s ability to displace fluids from dead-end structures. The regions circled by red dotted lines highlight that during waterflooding, the aqueous phase encountered significant flow resistance at constricted pore throats. This resistance could not be overcome regardless of the injection duration. Upon transitioning to surfactant flooding, the reduction in interfacial tension induces changes in flow dynamics, thereby lowering the mobilization pressure for crude oil. This process is accompanied by distinct emulsification and solubilization phenomena, where crude oil was dispersed into discrete droplets and partially solubilized in the aqueous phase. This dual mechanism enables the gradual formation of an oil bank, effectively accessing and producing trapped hydrocarbons from dead-end structures and narrow throats. The superior performance of the surfactant system is quantitatively confirmed by the significantly reduced residual oil saturation compared to waterflooding. Real-time visualization captured three key recovery processes: the continuous mobilization of emulsified droplets through flow channels, the complete displacement of oil in pore bodies following surfactant mixture flooding, and the advancement of the oil bank through pore–throat networks. These observations elucidate how viscoelastic surfactant solution overcame the capillary trapping mechanisms that limit the efficiency of waterflooding in heterogeneous porous media.

**Figure 14:**
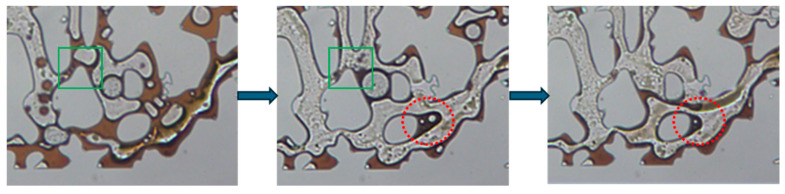
The viscoelastic phenomena during the surfactant flooding process in the pore–throat structure emulating realistic reservoir rock.

Figure 14 illustrates the unique oil displacement mechanism of the optimized viscoelastic surfactant mixture exhibiting gel-like properties. In the regions circled by red lines, the oil phase adhering to the surface was deformed under the influence of viscous and elastic forces and detached as droplets under shear stress. This process results in the mobilization and removal of the oil phase from the surface. In the regions circled by green lines, the small oil droplets formed after shearing collectively prompted the bulk oil within the narrow throats as they pass through. This phenomenon highlights the synergistic role of viscoelasticity in overcoming capillary trapping and improving interfacial flow dynamics.

**Figure 15:**
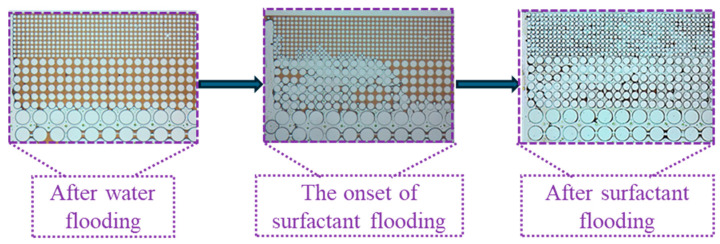
Mobility variation in the idealized pore–throat network with permeability contrast.

Owing to the planar heterogeneity of the structure, the fingering phenomenon of the injected fluid occurred during the waterflooding process, resulting in significant differences in recovery efficiency across various regions. As shown in Figure 15, formation water readily established dominant flow channels in the high-permeability zone. In contrast, the medium and low permeability zones exhibit higher capillary forces and greater flow resistance leading to the entrapment of a substantial amount of crude oil in these regions. Upon switching to surfactant solution flooding, the reduction in oil–water interfacial tension diminished capillary forces and decreased the resistance to oil flow. Consequently, crude oil in the medium permeability zone was mobilized followed by that in the low permeability zone after a period of displacement. The surfactant solution emulsified crude oil into smaller droplets, thus reducing the flow resistance of the oil.

**Figure 16:**
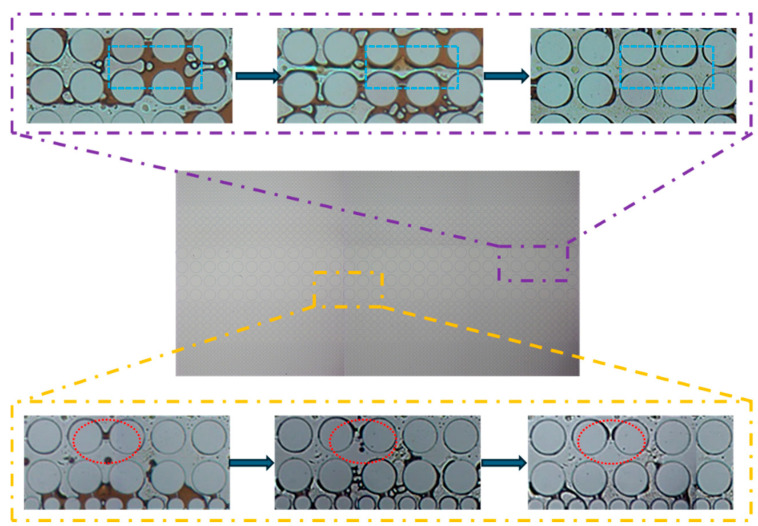
Images of the flooding experiment using the optimized surfactant formula in the idealized pore–throat network with permeability contrast.

Figure 16 shows that in an idealized pore–throat network with permeability contrast, the blue-lined area exhibits distinct emulsification and dissolution phenomena. In regions where surfactant solutions cannot flush directly, a noticeable emulsification and solubilization phenomenon occurs at the oil–water interface. Under the influence of surfactants, crude oil was either dissolved in water or emulsified into small droplets, which was then carried away by the displacement fluid. The red-lined area shows that crude oil adhering to a solid surface was significantly stretched and fragmented into small droplets due to the solution’s viscoelastic properties and the combined effects of elastic and viscous forces. These droplets were then carried away by the displacement fluid.

## 3. Conclusions

This study evaluated various combinations of anionic (SDS) and zwitterionic (OAB-30) surfactants in displacement tests, identifying a 0.2% SDS+0.6% OAB-30 mixture as the most effective formulation. This solution exhibited the highest viscosity, reduction interfacial tension, and favorable viscoelastic properties, thus resulting in superior oil displacement efficiency in dead-end pores. When applied to a micromodel simulating realistic reservoir rock, the optimized surfactant solution enhanced oil recovery by 28.46% over conventional water flooding. It effectively displaced crude oil from dead-end pores and throats. In a heterogeneous pore–throat network with permeability contrast, the surfactant increased oil recovery by 49.96% compared to water flooding. The gel-like properties of the solution improved sweep efficiency and mobility control, therefore mitigating the fingering in high-permeability zones, confirming its potential for enhanced recovery in complex reservoir systems.

## 4. Materials and Methods

### 4.1. Materials

**Figure 17:**
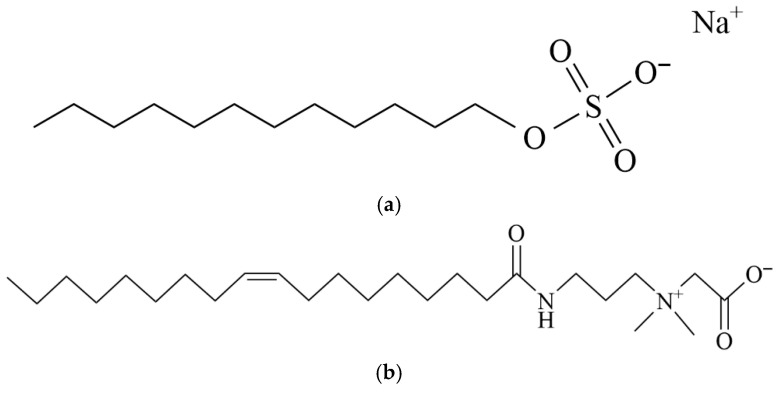
Chemical structure of the examined surfactants. (**a**) SDS; (**b**) OAB-30.

Oil was prepared by blending crude oil and high-viscosity liquid paraffin (Shanghai Macklin Biochemical Co., Ltd., Shanghai, China) at a 1:1 volume ratio. Simulated formation water (0.03% KCl, 0.07% MgCl_2_, 0.1% CaCl_2_, and 0.8% NaCl), sodium dodecyl sulfate (SDS, Tianjin Damao Chemical Reagent Factory, Tianjin, China), and oleic amide propyl betaine (OAB-30, 30%, Shandong Usolf Chemical Technology Co., Ltd., Linyi, China) were used in this study. Crude oil used in this work consists of 2.09% asphaltene, 22.78% resin, 4.27% aromatic hydrocarbons, and 70.86% saturated hydrocarbons. The chemical structures of the surfactants are shown in Figure 17. Six surfactant mixtures with varying compositions were prepared and the details are presented in Table 3.

### 4.2. Microfluidic Experiment Setup

**Figure 18:**
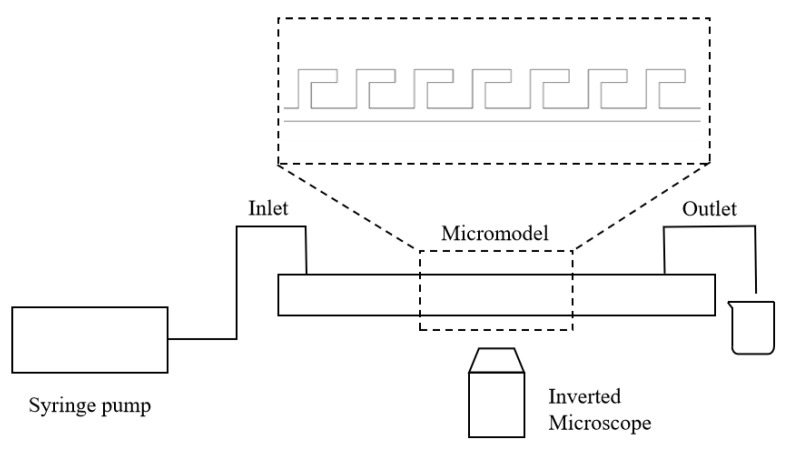
Schematic of the experimental setup.

The microfluidic system consists of a microinjection pump, an inverted microscope, a high-speed camera, and a computer. Microfluidic devices were fabricated using polydimethylsiloxane (PDMS) by mixing pre-polymer and curing agent (10:1 *w*/*w*) (SYLGARD 184 Silicone Elastomer Kit, Dow Corning, Midland, MI, USA) and curing on a silicon mold with a designed pattern at 65 °C. After demolding, fluidic ports were punched, followed by oxygen plasma bonding to a glass slide. The schematic of the experimental setup is shown in Figure 18. A microfluidic device was secured under the microscope equipped with the high-speed camera, which was connected to the computer. The microinjection pump was used to deliver fluids into the device at predetermined flow rates. The entire process was monitored and recorded in real-time via high-speed camera. Microfluidic devices with different pore–throat structures were used in this work.

### 4.3. Experimental Procedures

#### 4.3.1. Screening of Surfactant Formulations Using Microfluidic Device with Dead-End Structure

**Figure 19:**
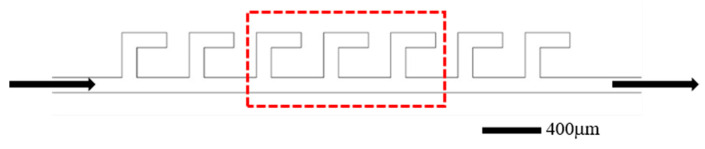
Schematic of the L-shaped dead-end pore structure. The arrows represent the flow direction.

The screening experiment was conducted using parallel L-shaped dead-end microfluidic structures, with the microfluidic device configuration illustrated in Figure 19. The experimental procedures are as follows: (1) prior to surfactant flooding, the fabricated microfluidic devices were initially saturated with simulated formation water, followed by oil injection until stable oil saturation was achieved, thereby simulating initial reservoir conditions; (2) subsequently, surfactant solutions were injected at a constant flow rate of 0.5 µL/min until a stable residual oil saturation was achieved. Images were taken periodically to calculate the oil saturation change and final oil recovery efficiency. The procedures were repeated for all six surfactant formulations. Three central structures (circled by red dotted lines in Figure 19) were selected for performance evaluation and the oil displacement efficiency in these structures was calculated and the average was taken as the oil displacement efficiency. The entire process was monitored using a microscope and high-speed camera to analyze fluid dynamics and displacement patterns within the microchannels.

**Figure 20:**
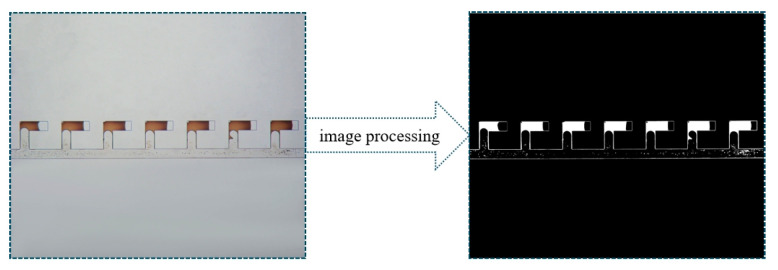
Demonstration of the image processing procedure.

The ImageJ software (NIH) was employed to process and analyze the images obtained during the experimental process. As illustrated in Figure 20, after binarization using the software, the white regions represent the crude oil. The area of the white regions was calculated using the software that was then used to represent the oil saturation given the micromodel depth was constant. Each image was measured three times, and the results exhibited a measurement error less than 3%.

#### 4.3.2. Microscopic Displacement Experiments with Microfluidic Devices with Different Pore–Throat Structures

**Figure 21:**
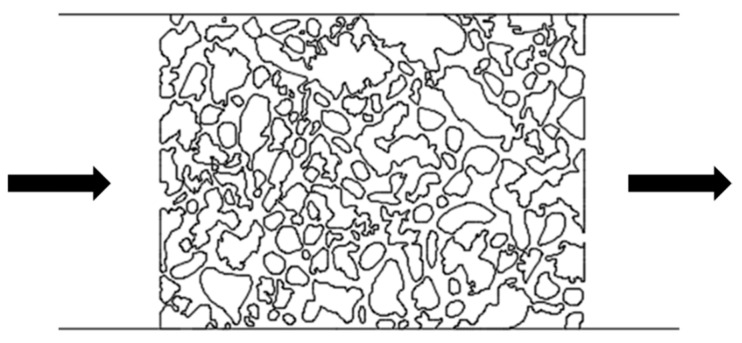
Schematic of the pore–throat structure emulating realistic reservoir rock. The arrows represent the flow direction.

**Figure 22:**
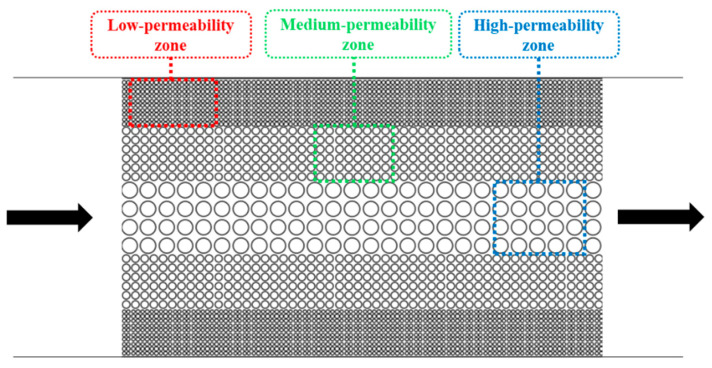
Schematic of the idealized pore–throat network with permeability contrast. The arrows represent the flow direction.

After screening surfactants using the device with L-shaped dead-end pores, microscopic displacement experiments were conducted using two distinct pore–throat configurations: pore–throat structure emulating realistic reservoir rock and idealized pore–throat network with permeability contrast as shown in Figure 21 and Figure 22. The experimental procedure involved initial saturation of the device with formation water and subsequent oil injection to establish irreducible water saturation and initial oil saturation, thus simulating the reservoir conditions. Formation water flooding was first performed. Upon stabilization of residual oil saturation, the injection fluid was switched to the optimized surfactant mixture solution with a constant injection rate of 0.5 µL/min. The same procedures were applied to both microfluidic devices.

#### 4.3.3. Determination of the Interfacial Tension

The interfacial tension between surfactant solutions and crude oil was measured using the spinning drop method (CNGTX, Shengwei Technology Co., Ltd., Beijing, China). Prior to testing, the centrifuge tube was thoroughly cleaned with organic solvent and deionized water to eliminate contaminant residues, followed by air drying. To minimize interference from impurities, the tubes were rinsed 2–3 times with small amounts of the surfactant solution. A droplet of crude oil was injected into the center of the solution using a microsyringe. Rotation speed was set at 5000 rpm and temperature was set at 30 °C. The measurement duration was 30 min, with automatic data acquisition every 2 min. Upon initiating the test procedure, the system continuously recorded dynamic interfacial tension variations over time. The stabilized segment of the resulting curve was selected as the final measurement value. To ensure statistical reliability, each sample was measured three times to minimize experimental errors.

#### 4.3.4. Determination of the Contact Angle

The contact angle was measured using the captive bubble method incorporating digital image analysis and contour fitting algorithms for enhanced measurement precision (KRÜSS DSA100, KRÜSS GmbH, Hamburg, Germany). The experimental procedures were as follows: (1) glass slides were aged in crude oil for 4 days to simulate reservoir conditions; (2) residual crude oil was thoroughly removed from the slides using anhydrous kerosene prior to contact angle measurement; (3) the sample cell was secured on the stage, and distilled water was added to fully submerge the substrate. The aged glass slide was then positioned within the cell; (4) a specially designed inverted needle was vertically inserted into the water, and carefully adjusted to dispense an oil droplet beneath the slide; the diameter of the oil droplets was approximately 3–5 mm and capture time was within 15 s. (5) The camera’s focus, lighting, and position were optimized to ensure clear visualization of the submerged oil droplet’s contour; (6) the droplet image was captured and analyzed to determine the initial contact angle. Following the initial measurement, the glass slide was subjected to an additional 4-day aging process in the surfactant solution, after which the contact angle was measured again to evaluate wettability alteration.

#### 4.3.5. Determination of the Viscosity of Surfactant Mixtures

The viscosity measurements in this study were conducted using a rotational viscometer (ROTAVISC me-vi, IKA, Staufen, Germany). Prior to testing, the instrument was carefully leveled and preheated to a stabilized temperature of 30 °C using the integrated temperature control system. With the rotational speed set to 170 rpm, viscosity readings were recorded only after achieving stable measurement values. This standardized procedure was repeated for all fluid samples under identical experimental conditions to ensure measurement consistency and reliability.

#### 4.3.6. Determination of the Viscoelasticity of Surfactant Mixtures

The viscoelastic properties of six surfactant solutions were characterized using a rheometer (Anton Paar MCR 102, Anton Paar GmbH, Graz, Austria). Steady-state shear tests were first conducted by applying controlled shear rates to obtain strain versus storage modulus (G’) and loss modulus (G’’) curves. The optimal strain value was determined by identifying the plateau region where both moduli remained stable. Subsequent dynamic oscillatory tests were performed at this stabilized strain level across a frequency range of 0.1–100 rad/s. The resulting angular frequency versus G’ and G’’ curves were recorded to fully characterize the viscoelastic behavior of each polymer solution under dynamic conditions.

## Figures and Tables

**Table 1:** Interfacial tension between crude oil and varying surfactant mixtures.

Solution Composition	Interfacial Tension (mN/m)	
	Mean Value	Standard Deviation
0.2%SDS+0.2%OAB-30	0.376	0.020
0.2%SDS+0.4%OAB-30	0.444	0.011
0.2%SDS+0.6%OAB-30	0.395	0.023
0.4%SDS+0.2%OAB-30	1.539	0.076
0.4%SDS+0.4%OAB-30	0.521	0.016
0.4%SDS+0.6%OAB-30	0.430	0.021

**Table 2:** Different anionic–zwitterionic surfactant formulations and the increase in oil recovery efficiency.

No.	Solution Composition	Increase in Oil Recovery Efficiency, %
1	Sodium alkyl glucosyl hydroxypropyl sulfonate + octadecyl betaine [38]	20
2	Sodium dodecyl benzene sulfonate + cocoamidopropyl sulfonate betaine [39]	18.5
3	Sodium alkylphenol polyoxyethylene ether carboxylate + dodecyl dimethyl hydroxypropyl sulfobetaine [40]	14
4	Nonylphenol polyoxyethylene ether carboxylate + oleic amide propyl betaine [41]	14.8

**Table 3:** Composition of surfactant solutions.

No.	Solution Composition	SDS (mmol/L)	OAB-30 (mmol/L)
1	0.2%SDS+0.2%OAB-30	6.94	4.52
2	0.2%SDS+0.4%OAB-30	6.94	9.04
3	0.2%SDS+0.6%OAB-30	6.94	13.55
4	0.4%SDS+0.2%OAB-30	13.87	4.52
5	0.4%SDS+0.4%OAB-30	13.87	9.04
6	0.4%SDS+0.6%OAB-30	13.87	13.55

## Data Availability

The authors confirm that the data are provided within the article.
